# DHHC protein family targets different subsets of glioma stem cells in specific niches

**DOI:** 10.1186/s13046-019-1033-2

**Published:** 2019-01-18

**Authors:** Xueran Chen, Lei Hu, Haoran Yang, Huihui Ma, Kaiqin Ye, Chenggang Zhao, Zhiyang Zhao, Haiming Dai, Hongzhi Wang, Zhiyou Fang

**Affiliations:** 1 Anhui Province Key Laboratory of Medical Physics and Technology; Center of Medical Physics and Technology, Hefei Institutes of Physical Science, Chinese Academy of Sciences, No. 350, Shushan Hu Road, Hefei, 230031 Anhui China; 2 University of Science and Technology of China, No. 96, Jin Zhai Road, Hefei, 230031 Anhui China; 3 Hefei Cancer Hospital, Chinese Academy of Sciences, No. 350, Shushan Hu Road, Hefei, Anhui, Hefei, 230031 Anhui China; 4 Key Laboratory of Ion Beam Bioengineering, Hefei Institutes of Physical Science, Chinese Academy of Sciences, No. 350, Shushan Hu Road, Hefei, 230031 Anhui China; 5 Department of Radiation Oncology, First Affiliated Hospital, Anhui Medical University, No. 218, Jixi Road, Hefei, 230031 Anhui China

**Keywords:** DHHC protein, BMI1, Glioma stem cell, Glioblastoma

## Abstract

**Background:**

Glioblastomas (GBM) comprise different subsets that exhibit marked heterogeneity and plasticity, leading to a lack of success of genomic profiling in guiding the development of precision medicine approaches against these tumors. Accordingly, there is an urgent need to investigate the regulatory mechanisms for different GBM subsets and identify novel biomarkers and therapeutic targets relevant in the context of GBM-specific niches. The DHHC family of proteins is associated tightly with the malignant development and progression of gliomas. However, the role of these proteins in the plasticity of GBM subsets remains unclear.

**Methods:**

This study utilized human glioma proneural or mesenchymal stem cells as indicated. The effects of DHHC proteins on different GBM subsets were investigated through in vitro and in vivo assays (i.e., colony formation assay, flow cytometry assay, double immunofluorescence, western blot, and xenograft model). Western blot, co-immunoprecipitation, and liquid chromatograph mass spectrometer-mass spectrometry assays were used to detect the protein complexes of ZDHHC18 and ZDHHC23 in various GBM subtypes, and explore the mechanism of DHHC proteins in targeting different subsets of GSCs in specific niches.

**Results:**

ZDHHC18 and ZDHHC23 could target the glioma stem cells of different GBM subsets in the context of their specific niches and regulate the cellular plasticity of these subtypes. Moreover, mechanistic investigations revealed that ZDHHC18 and ZDHHC23 competitively interact with a BMI1 E3 ligase, RNF144A, to regulate the polyubiquitination and accumulation of BMI1. These events contributed to the transition of glioma stem cells in GBM and cell survival under the stressful tumor microenvironment.

**Conclusions:**

Our work highlights the role of DHHC proteins in the plasticity of GBM subsets and reveals that BMI1 represents a potential therapeutic target for human gliomas.

**Electronic supplementary material:**

The online version of this article (10.1186/s13046-019-1033-2) contains supplementary material, which is available to authorized users.

## Background

Current therapies for human glioblastomas (GBM), the most common and deadly kind of primary intracranial tumor, are only palliative [1, 2]. Because of marked intra- and intertumoral heterogeneity, as well as cellular and molecular complexities, GBM do not respond to therapies targeting single molecular pathways [3, 4]. GBM contain stem cell-like tumor cells (GSCs), which are non-uniformly distributed in the tumors and abundant in the hypoxic and perivascular niches [5, 6]. This suggests that GSCs critically interact with the tumor microenvironment. In particular, previous reports indicate that the maintenance of GSCs can be promoted by microenvironmental stressors, such as nutrient restriction, hypoxia, and acids [5, 7].

With recent improvements in gene technology, molecular signatures are becoming increasingly essential for the classification of gliomas [8, 9]. Four molecular sub-classes of GBM, namely classical, proneural, neural, and mesenchymal, were identified in patients using whole-genome studies based on The Cancer Genome Atlas (TCGA) [10, 11]. The mesenchymal subtype can be distinguished from the other subtypes owing to its particularly aggressive characteristic [12, 13]. Because the transcriptional groups of GBM are plastic, there exists for example a transition of proneural group to mesenchymal group after cytotoxic therapy, and genomic profiling has not been successful in guiding the development of precision medicine for GBM [14, 15]. There is, therefore, an urgent need for investigating the regulatory mechanisms responsible for different GBM subsets and identifying novel biomarkers and therapeutic targets for these subsets considering the context of GBM-specific niches.

As a major regulatory component of the complex of polycomb repressive complex 1 required for the activity of H2A-K119 ubiquitin E3 ligase, BMI1 polycomb ring finger oncogene (BMI1) serves as a potent inducer of neural progenitor cell proliferation and neural stem cell self-renewal during growth and is, thus, involved in maintaining the homeostasis of adult tissues [16, 17]. Notably, the gene for BMI1 is aberrant at the chromosomal level in gliomas, contributing to the pathogenesis of tumors; BMI1 might also initiate cells that promote the undifferentiated state of GBM cells [18, 19]. Furthermore, BMI1 regulates and binds to the promoters of many genes, such as transforming growth factor-β (TGFβ), which is intricately associated with the mesenchymal phenotype [20]. Indeed, the expression of BMI1 is enhanced in the cells of mesenchymal GBM relative to that in proneural cells [21]. Nonetheless, the regulation of BMI1 expression in various GBM subtypes and the role of this protein in the transition of molecular subtypes are poorly understood.

Several recent studies have indicated that the DHHC (Asp-His-His-Cys)-S-acyltransferase protein family and its substrates have pivotal roles in tumorigenesis, especially in the development of malignancy and progression of glioma [22–24]. Here, we demonstrate that ZDHHC18 and ZDHHC23, which are members of the DHHC family, target different GSC subsets in specific niches and regulate the plasticity of cells in these subtypes by influencing the polyubiquitination of BMI1. These findings highlight the importance of BMI1 expression pattern regulation by DHHC family members in the plasticity of GBM GSCs. They also suggest that DHHC proteins and BMI1 may serve as potential therapeutic targets for human gliomas.

## Methods

### Human GBM specimens

Paraffin-embedded archival primary GBMs were obtained from the Department of Pathology of the Cancer Hospital of Hefei Institutes of Physical Science, Chinese Academy of Sciences (CAS) (Anhui, China). The surgeon selected only those tumors with significant quantities of peritumor regions, leading edge, and infiltrating tumor regions as well as necrotic avascular regions in the center of the tumor, as described previously [21]. These distinct specimens from different anatomic regions of each tumor were then processed separately and analyzed as indicated below. The study protocols were approved by the Institutional Review Board of the Cancer Hospital of Hefei Institutes of Physical Science, CAS, and written informed consent was obtained from patients based on the Declaration of Helsinki.

### Cell culture and plasmids

The subtype-characterized GSCs (PN12, PN16, PN19, MES23, MES27, and MES29) were isolated from surgical specimens as reported in a previous study [25]. The normal neural progenitor cells (NPCs) were obtained from Lonza (Amagazaki, Japan) in 2015. NPCs and GSCs were maintained in Neurobasal medium with B27 (without vitamin A, Invitrogen, Carlsbad, CA), basic fibroblast growth factor (20 ng/ml), and epidermal growth factor (20 ng/ml). Trypan blue staining followed by fluorescence-activated cell sorting (Beckman Coulter, Indianapolis, IN) analysis was used to evaluate the viability of the newly formed GSC spheres, and bromodeoxyuridine (BrdU; Sigma-Aldrich, St. Louis, MO) incorporation was measured according to manufacturer instruction. Functional assays including prospective enrichment of stem cell marker expression and sphere formation were used for validation. Molecular subtyping of these GBM cells was performed by either expression array or RNA sequencing (Additional file 1: Figure S1). All the cell lines used in this study were subjected to STR analysis and tested for mycoplasma, most recently in August 2018.

Lentiviral plasmids for targeting ZDHHC18 (shRNA, TF307371; LentiORF, RC209313L3), ZDHHC23 (shRNA, TL300344; LentiORF, RC207272L3), and non-specific control sequence (shCNTRL) were purchased from Origene (Rockville, MD). Lentiviral particles were produced in HEK293FT cells with PAX2 and PMD2G helper plasmids (Addgene, Cambridge, MA) in stem cell medium.

### Quantitative RT-PCR

Total RNA was isolated using an RNeasy kit (Qiagen, Venlo, The Netherlands) and was reverse-transcribed into cDNA using the SuperScript III First-Strand Synthesis kit (Invitrogen). Real-time PCR was performed on an Applied Biosystems 7300 cycler (Foster City, CA) using SYBR Green PCR MasterMix (Invitrogen). Each sample was prepared in triplicate and the levels of target gene expression were calculated using the 2^−ΔΔCt^ method, with *GAPDH* serving as the internal control. The sequences of gene-specific primers used in the study were as follows: *ZDHHC18*, 5′-CTT CTT CGT CAT GAG CTG CC-3′ and 5′-CTT CTT CGT CAT GAG CTG CC-3′; *ZDHHC23*, 5′-GTC GGG CAG TCT CAA CAA TC-3′ and 5′-TCC TCA CAC AGA TGC CAC AT-3′; *DLL3*, 5′-CCA GGT CCT TTG AAT GCA CC-3′ and 5′-CAG TTG GAG CCT TGG AAA CC-3′; *OLIG2*, 5′-CGT CTC AAG ATC AAC AGC CG-3′ and 5′-CGT AGA TCT CGC TCA CCA GT-3′; *ASCL1*, 5′-GCG GCC AAC AAG AAG ATG AG-3′ and 5′-AGT CGT TGG AGT AGT TGG GG-3′; *CD133*, 5′-TTC TTG ACC GAC TGA GAC CC-3′ and 5′-CCA AGC ACA GAG GGT CAT TG-3′; *SOX2*, 5′-TGA TGG AGA CGG AGC TGA AG-3′ and 5′-GCT TGC TGA TCT CCG AGT TG-3′; *CD44*, 5′-CGC CAA ACA CCC AAA GAA GA-3′ and 5′-TTC CTG CTT GAT GAC CTC GT-3′; *CHI3L1*, 5′-GTC CAT AGA ATC CTC GGC CA-3′ and 5′-ATG GCA TTG GTG AGA GGG AA-3′; *TIMP1*, 5′-CCT TCT GCA ATT CCG ACC TC-3′ and 5′-GTA TCC GCA GAC ACT CTC CA-3′; *TGFB1*, 5′-CTT TCC TGC TTC TCA TGG CC-3′ and 5′-TCC AGG CTC CAA ATG TAG GG-3′; and *GAPDH*, 5′-AGG TCG GAG TCA ACG GAT TT-3′ and 5′-TGA CGG TGC CAT GGA ATT TG-3′.

### Immunoprecipitation and western blot analysis

Protein–protein interaction and BMI1 polyubiquitination were detected using a Pierce Crosslink magnetic IP and Co-IP kit (Thermo Scientific, Waltham, MA). For the ubiquitination assays, cells were treated with Lactacystin (5 μM or 10 μM; Sigma, St. Louis, MO) for 5 h before collection. The immunoprecipitated and co-immunoprecipitated proteins were analyzed by SDS-PAGE and western blotting.

Equal amounts of cell lysate were resolved by SDS-PAGE and transferred onto a nitrocellulose blotting membrane (Pall) that was subsequently exposed to primary antibodies against ZDHHC18 (ab154790, Abcam, Cambridge, UK), ZDHHC23 (ab121513, Abcam), BMI1 (#6964, Cell Signaling Technology, Danvers, MA), RNF144A (ab75054, Abcam), H2AK119Ub (#8240, Cell Signaling Technology), and β-actin (#3700, Cell Signaling Technology). The membranes were then treated with the corresponding horseradish peroxidase-conjugated secondary antibodies (Sigma) and the bands were detected using enhanced chemiluminescence (Pierce, Rockford, IL).

### Cell viability assays

The viability of cells was determined using the CellTiter-Glo Luminescent Cell Viability Assay kit (Promega, Madison, WI), according to manufacturer instruction, after 48 h exposure to different conditions, including low (0.45 g/l) glucose, hypoxia (1% oxygen), and BMI1 inhibitor (PTC596, PTC Therapeutics, South Plainfield, NJ).

### In vitro tumorsphere formation and colony formation assay

For suspension culture/tumorsphere formation, 500 cells were seeded in 6 well plates containing 2 ml of complete neurobasal medium and treated with BMI1 inhibitors for 3 days or left untreated. After 10 days, the tumorspheres were measured and analyzed.

For colony formation assays, 500 cells were plated in a 10 cm plate in standard growth medium and treated with BMI1 inhibitors for 3 days or left untreated. After 2 weeks of growth, the cells were fixed and cell colonies were counted and analyzed.

### Flow cytometry

The specimens from different anatomic regions of glioma tumors were enzymatically dissociated into single cells and incubated with anti-ZDHHC18 (or ZDHHC23), and CD133 (or CD44) antibodies. Thereafter, the cells were treated with corresponding secondary antibodies and analyzed with Beckman Coulter Cyto FLEX and CXP analysis software (Beckman Coulter Inc., Miami, FL).

### Animal experiments

Animal experiments were performed according to the guidelines of the Animal Use and Care Committees at Hefei Institutes of Physical Science, CAS (Anhui, China). All the mice were randomly assigned to appropriate treatment groups. For the implantation study, 500 PN12 or 500 MES23 (or shRNA-ZDHHC18 MES23) GSCs were implanted intracranially into 6 week old NOD SCID gamma (NSG) mice. After 1 week, a BMI1 inhibitor (PTC596) was administered orally by gavage. Finally, the tumor-bearing mice were sacrificed and analyzed.

### In silico analyses and statistical analysis

The web-accessible Gene Expression across Normal and Tumor Tissue (GENT) database (http://medical-genome.kribb.re.kr/GENT) was utilized to assess the expression patterns of ZDHHC18 and ZDHHC23 in the tissues of brain cancer. The collected in silico resources including transcript microarray data, RNA sequencing, patient survival, and anatomic information of patient samples were downloaded from The Cancer Genome Atlas (TCGA) (https://tcga-data.nci.nih.gov/tcga) and Ivy GAP (http://glioblastoma.alleninstitute.org) websites. To conduct the survival analysis, a Kaplan–Meier plotter (http://kmplot.com) was used.

All grouped data are presented as the means ± standard error of the mean (SEM). The significance between groups was analyzed by one-way ANOVA or Student’s *t*-test using GraphPad Prism software (LaJolla, CA). All the experiments were repeated for each specimen in at least three biological duplicates. The criterion for significance (*p* value) was set as mentioned in the figures.

## Results

### Up-regulation of ZDHHC18 and ZDHHC23 is associated with increasing tumor grade in gliomas

**Fig. 1:**
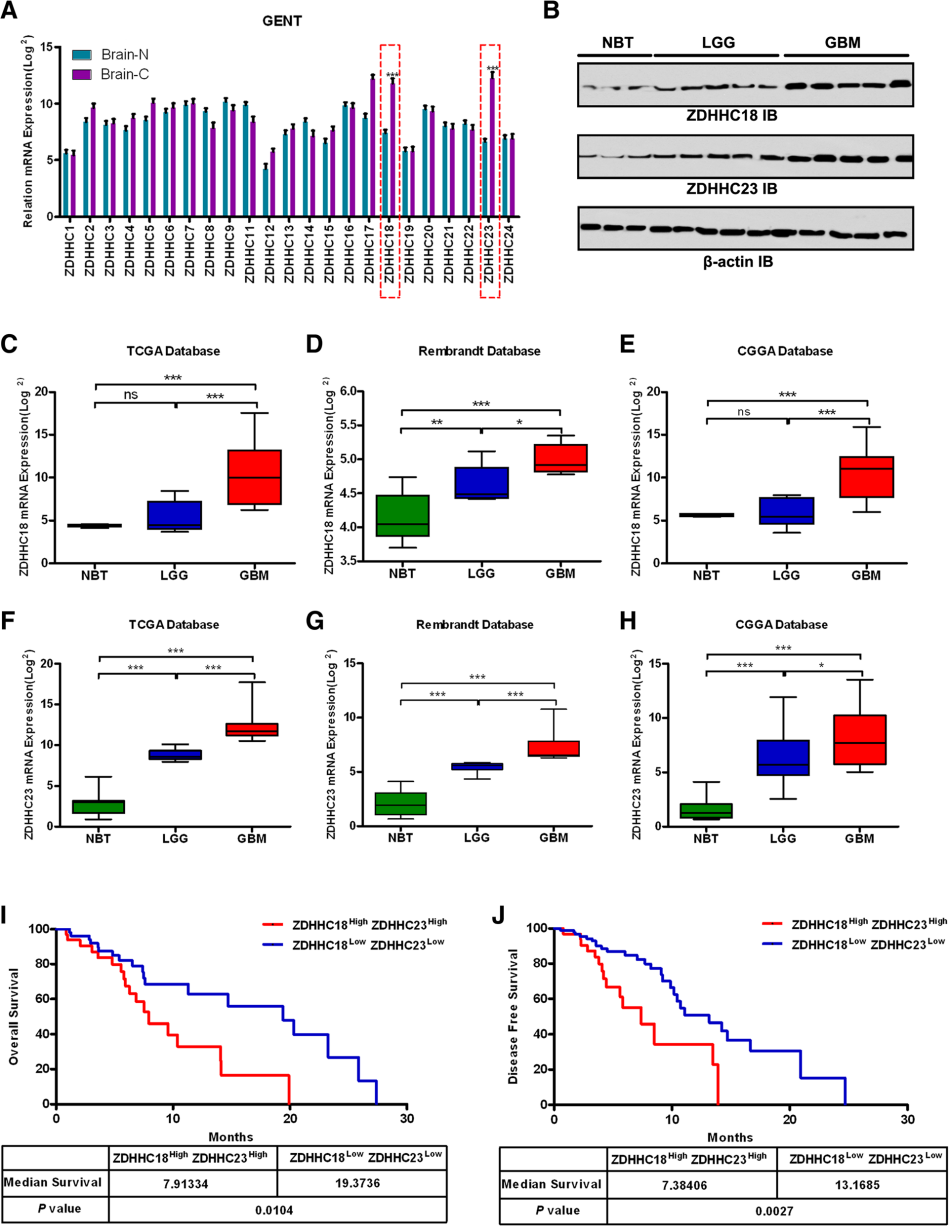
Expression of ZDHHC18 or ZDHHC23 is associated with tumor grade in gliomas. **a** Public data retrieved from the GENT database indicate that the expression levels of ZDHHC18 and ZDHHC23 are higher in brain cancer tissues (C) than those in normal brain tissues (N). The data were downloaded to normalized log2 value for each gene in the database and the graph was re-drawn in R program. (***, *p* < 0.001; Brain-N: *n* = 176, brain-C: *n* = 2357). **b** Expression levels of ZDHHC18 and ZDHHC23 as detected by western blot analysis in LGG, and GBM gliomas and normal brain tissues. β-actin was used as a loading control. NBT, normal brain tissue; LGG, low grade glioma; GBM, glioblastoma multiforme. **c**-**e** Quantification of *ZDHHC18* mRNA expression levels in gliomas in TCGA (**c**), Rembrandt (**d**), and CGGA (**e**) datasets (*ns*, not significant; *, *p* < 0.05; **, *p* < 0.01; ***, *p* < 0.001). **f**-**h** Quantification of *ZDHHC23* mRNA expression levels in gliomas in TCGA (**f**), Rembrandt (**g**), and CGGA (**h**) datasets (*, *p* < 0.05; ***, *p* < 0.001). **i** and **j** Cumulative overall survival (**i**) and disease-free survival (**j**) of patients with GBM and low/high co-expression levels of ZDHHC18/ZDHHC23 (based on median expression levels of ZDHHC18 and ZDHHC23, respectively) estimated using the Kaplan–Meier method and compared with the log-rank test in the same set of patients (*n* = 32; *, *p* < 0.05; ***, *p* < 0.001)

To study the roles of DHHC proteins in gliomas, we first analyzed in silico data from GENT (Fig. 1a). Significant up-regulation of ZDHHC18 and ZDHHC23, especially the latter, was observed in a comparative analysis of 176 normal brain tissues and 2357 glioma tissues. Consistent with these results, the protein levels of ZDHHC18 and ZDHHC23 in gliomas were found to be elevated relative to those in the normal brain tissue and positively correlated with the degree of malignancy (Fig. 1b). We further validated these findings using three additional published datasets: TCGA, the National Cancer Institute Repository for Molecular Brain Neoplasia Data (REMBRANDT), and the Chinese Glioma Genome Atlas (CGGA) (Fig. 1c-h). In these datasets, ZDHHC18 or ZDHHC23 was also found to be highly expressed in the GBM samples compared to that in the low-grade gliomas (LGGs) and normal brain tissues. However, no significant differences in ZDHHC18 expression were observed between LGGs and normal tissues in the TCGA and CGGA databases.

We then investigated the relationship between clinicopathological characteristics of patients and ZDHHC18 and ZDHHC23 expression levels in 90 glioma tissues (Additional file 1: Table S1). The high expression of ZDHHC18 (median value) was statistically associated with patient Karnofsky Performance Scale (KPS) value (< 80, *p* < 0.05). The high levels of expression of ZDHHC18 and ZDHHC23 were related to the tumor grade (IV, *p* < 0.01 for ZDHHC18; IV, *p* < 0.01 for ZDHHC23). Notably, ZDHHC18 up-regulation was associated with the mesenchymal GBM subtype (*p* < 0.01) and ZDHHC23 expression was related to the proneural subtype (*p* < 0.01). Some molecular genetic features including *IDH1/2* mutation, *MGMT* promoter methylation, co-deletion of 1p/19q, TERT loss, and *ATRX* mutation have been reported to be associated with favorable prognosis in gliomas. We therefore investigated whether the expression of ZDHHC18 and ZDHHC23 correlated with these characteristics. The patients with wild type *IDH1* exhibited higher expression of ZDHHC18 than those with mutated *IDH1* (*p* < 0.01). The low expression level of ZDHHC18 was also associated with other molecular characteristics including 1p/19q co-deletion, loss of *TERT*, and mutated *ATRX* in tumors (*p* < 0.01; respectively). Moreover, low level of ZDHHC23 was also associated with 1p/19q co-deletion (*p* < 0.01), loss of *TERT* (*p* < 0.05), and mutated *ATRX* (*p* < 0.01) in the glioma tumors. The prognostic value of ZDHHC18 and ZDHHC23 co-expression in the overall survival (OS) and disease free survival (DFS) of patients with glioma was examined using Kaplan–Meier survival curves (Fig. 1i and j). High coexpression levels of ZDHHC18 and ZDHHC23 (> median value) had a significantly worse prognosis than low co-expression levels in the patients with GBM (7.91 vs. 19.37 months for OS, *p* < 0.05; 7.38 vs. 13.17 months for DFS, *p* < 0.01). Thus, expression levels of ZDHHC18 and ZDHHC23 were positively correlated with the increasing tumor grade, both in the publicly available databases and in our cohort of primary tumor specimens, and might, therefore, constitute novel prognostic biomarkers for gliomas.

### Association of ZDHHC18 and ZDHHC23 expression levels with GBM subgroups in patients with gliomas

**Fig. 2:**
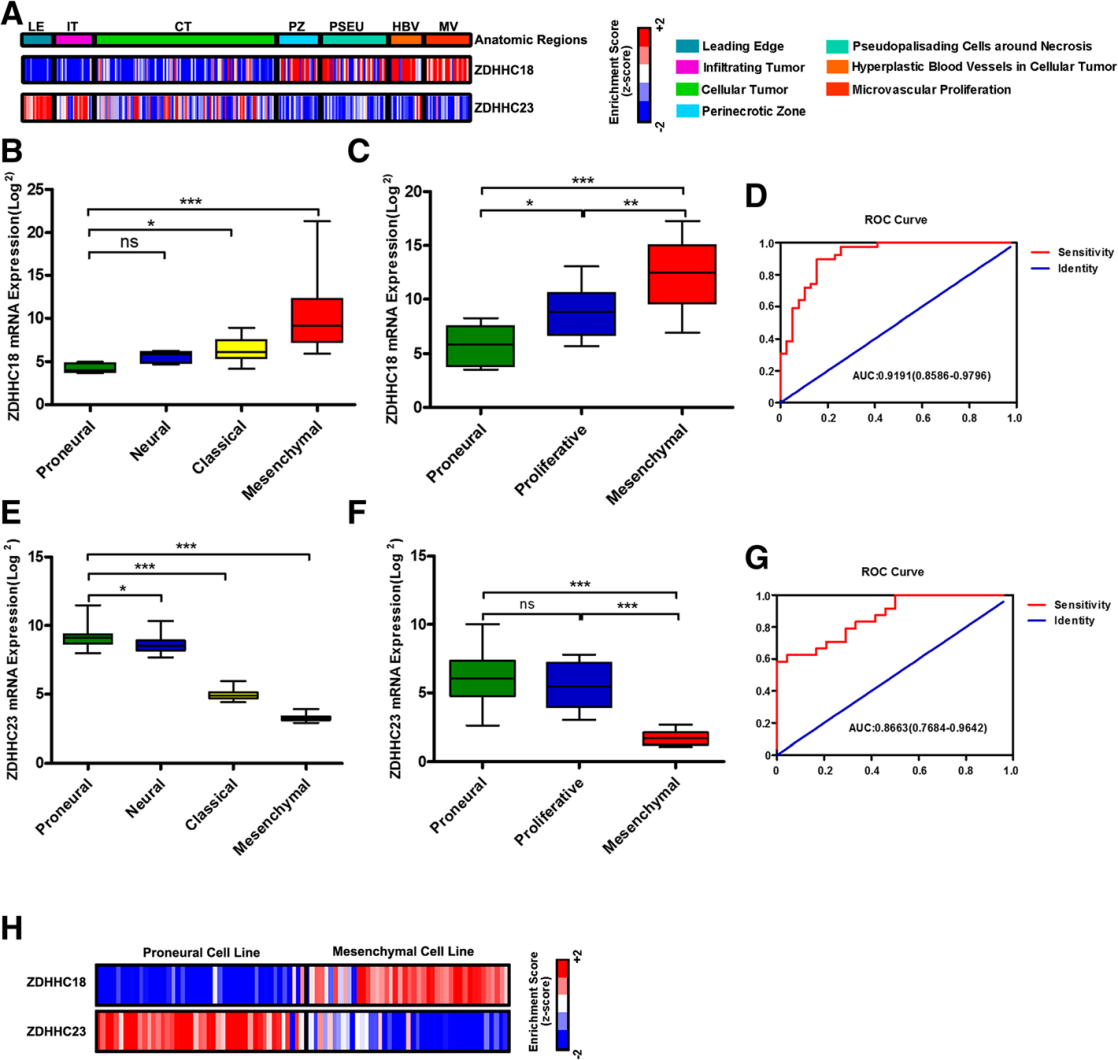
Expression of ZDHHC18 or ZDHHC23 is associated with glioblastoma (GBM) subgroups in patients with glioma. **a** Heatmap showing the expression pattern of ZDHHC18 and ZDHHC23 as indicated by Z-scores in distinct anatomic regions of GBM tissues from the Ivy GAP database (RNA-sample, *n* = 122; patient, *n* = 10). The corresponding histological feature for each RNA-sample is labeled as follows: leading edge (LE); infiltrating tumor (IT); cellular tumor (CT); perinecrotic zone (PZ); pseudopallisading cells around necrosis (PSEU); hyperplastic blood vessels in cellular tumor (HBV); microvascular proliferative region (MV). **b** and **c** Quantification of GBM subtype-specific ZDHHC18 expression in the TCGA and GSE4271 datasets. Log2-transformed values of the expression levels of *ZDHHC18* mRNA are listed on the Y-axis. Error bars represent the SEM. **d** Receiver operating characteristic (ROC) curve showing sensitivity of ZDHHC18 as a marker to distinguish patients with mesenchymal from non-mesenchymal subtype GBM. **e** and **f** Quantification of GBM subtype-specific ZDHHC23 expression in the TCGA and GSE4271 datasets. Log2-transformed expression of *ZDHHC23* mRNA levels is listed on the Y-axis. Error bars represent the SEM. **g** ROC curve showing the sensitivity of ZDHHC23 as a marker to distinguish patients with proneural from non-proneural subtype GBM. **h** Heatmap showing the expression pattern of ZDHHC18 and ZDHHC23 as indicated by Z-scores in the proneural and mesenchymal glioma cell lines from the GENT database (RNA-sample *n* = 266)

GBM display marked heterogeneity both between and within tumors, with these heterogeneities arising from variations in genetics, epigenetic cell state, and the microenvironment [3, 26]. We interrogated the Ivy Glioblastoma Atlas Project (Ivy GAP) database, which contains data from 55 glioblastomas regionally microdissected with RNA sequencing (http://glioblastoma.alleninstitute.org/), and investigated the relationship between the expression of ZDHHC18 and ZDHHC23 and their anatomical distribution. The ZDHHC18 expression level was increased in perinecrotic and microvascular proliferative regions, and that of ZDHHC23 was associated with the leading edge and infiltrating tumor regions in GBM (Fig. 2a). As reported, the leading edge and infiltrating tumor regions expressed a proneural signature, whereas perinecrotic and microvascular proliferative regions expressed a mesenchymal signature [21]. Confirming our findings from the Ivy GAP results, the expression level of ZDHHC18 was found to be high in the mesenchymal subtype in the TCGA and GSE4271 databases and low in the proneural subtype (Fig. 2b and c; Additional file 1: Table S1), whereas ZDHHC23 expression was increased in the proneural subtype compared to that in the mesenchymal subtype (Fig. 2e and f; Additional file 1: Table S1).

Moreover, the receiver operating characteristic curve further showed sensitivity of ZDHHC18 as a marker in distinguishing patients with GBM exhibiting mesenchymal subtype from those with non-mesenchymal subtype (Fig. 2d), and that of ZDHHC23 as a marker of proneural subtype (Fig. 2g). Consistent with these results, there was a significant inverse correlation of ZDHHC18 expression level, as a marker for mesenchymal subtype, with ZDHHC23 expression, as a marker for the proneural subtype, in proneural and mesenchymal cell lines (Fig. 2h). We therefore demonstrated that the expression of ZDHHC18 and ZDHHC23 is associated with distinct anatomical distribution in GBM, and is separately defined for the mesenchymal and proneural features.

### ZDHHC18 and ZDHHC23 target different subsets of GSCs in specific niches

**Fig. 3:**
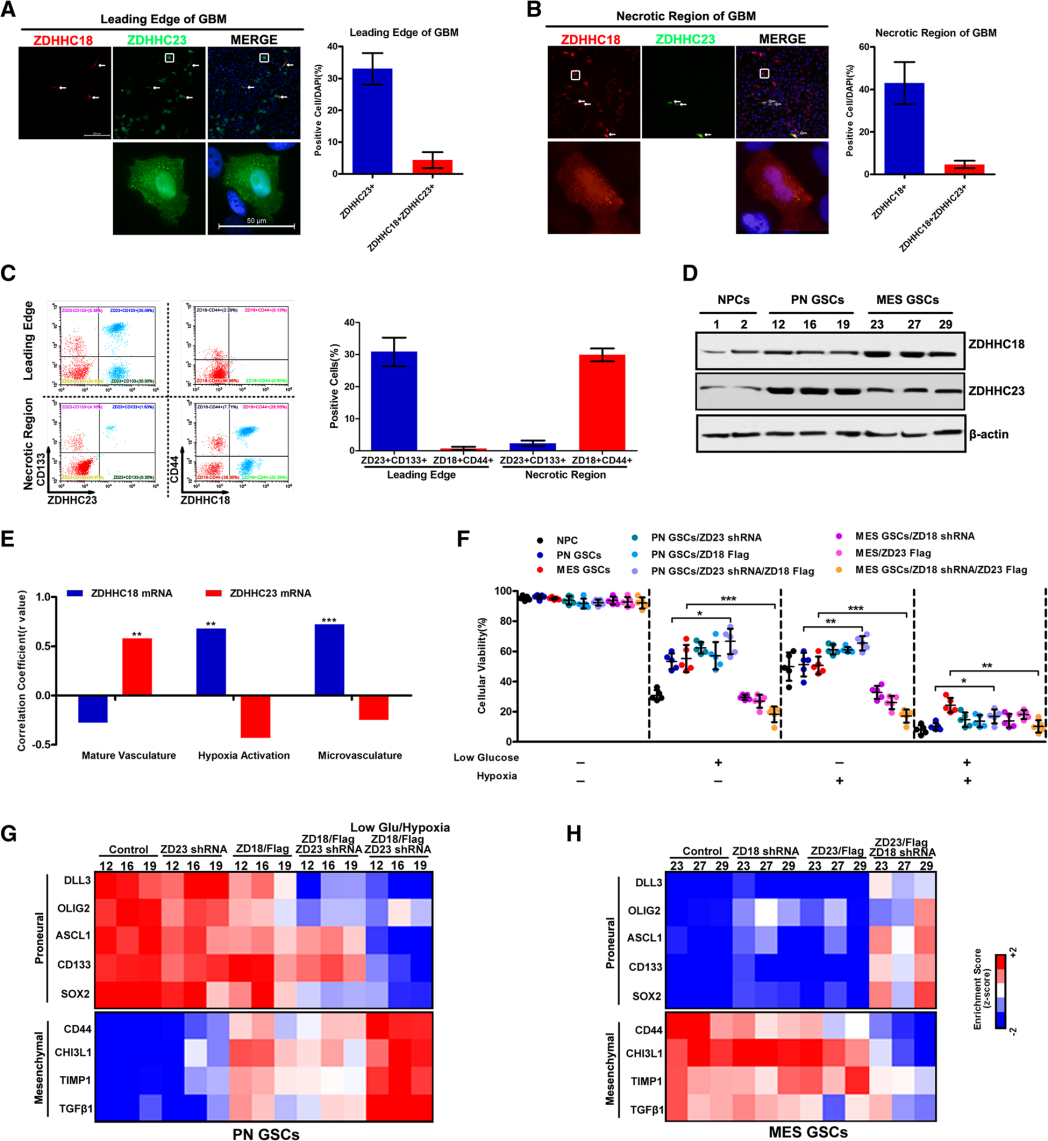
ZDHHC18 and ZDHHC23 target different subsets of glioma stem cells (GSCs) in specific niches. **a, b** Immunofluorescence images showing ZDHHC18 (red) and ZDHHC23 (green) positive locations and cells in the leading edge (**a**) and neurotic (**b**) regions of the glioblastoma (GBM) samples. Scale bar (upper image): 200 μm; (lower image, inset box): 50 μm (white). Quantification of ZDHHC23 positive or ZDHHC18 and ZDHHC23 positive cells in three fields is presented. Error bars represent the SEM. **c** Results of flow cytometry analysis showing the relationship between the expression of ZDHHC18 (or ZDHHC23) and stem cell marker CD133 (or CD44) in the leading edge and neurotic regions of the GBM samples. Quantification of ZDHHC18/CD133, ZDHHC23/CD133, ZDHHC18/CD44, and ZDHHC23/CD44 positive cells in three fields is presented. Error bars represents the SEM. **d** Expression levels of ZDHHC18 and ZDHHC23 detected by western blot analysis in neural progenitor cells (NPC1 and NPC2), proneural GSCs (PN12, PN16, and PN19), and mesenchymal GSCs (MES23, MES27, and MES29). β-actin was used as a loading control. **e** Bar graph showing Pearson coefficients of correlation between *ZDHHC18* (blue) or *ZDHHC23* (red) mRNA expression and the mature vasculature, microvasculature, and hypoxia activation signatures in 12 GSC and one NPC culture (**, *p* < 0.01; ***, *p* < 0.001). **f** Cell viability of NPC1, proneural GSCs (PN12), mesenchymal GSCs (MES23), or proneural (or mesenchymal) GSCs transfected with indicated plasmids was determined under baseline, low-glucose, hypoxia, or conditions with a combination of these stresses. Data are presented as means ± SEM (*, *p* < 0.05; **, *p* < 0.01; ***, *p* < 0.001). **g** and **h** Heatmap showing the molecular subtype marker expression in proneural GSCs (PN12, PN16, and PN19), mesenchymal GSCs (MES23, MES27, and MES29), and proneural (or mesenchymal) GSCs transfected with indicated plasmids under baseline or low-glucose/hypoxia stress. Proneural markers: DLL3, OLIG2, ASCL1, CD133, and SOX2; mesenchymal markers: CD44, CHI3L1, TIMP1, and TGFβ1. Z-scores were calculated from the ΔCt values obtained in the qPCR analysis

The different anatomical regions of glioblastoma were analyzed by immunofluorescence using antibodies against ZDHHC18 and ZDHHC23, which demonstrated that both ZDHHC18 and ZDHHC23 were mostly localized in the nucleus and cytoplasmic vesicles around the nucleus. Notably, ZDHHC23 was localized in the leading edge (Fig. 3a), whereas ZDHHC18 expression was confined to the necrotic region (Fig. 3b). Consistent with these results, the flow cytometry data revealed that ZDHHC23 was exclusively co-expressed with the proneural GSC marker, CD133, in the leading edge, and ZDHHC18 was tightly associated with the mesenchymal GSC marker, CD44, in the necrotic region of GBM (Fig. 3c). In contrast, only a small fraction of ZDHHC18-expressing cells were present in the CD133-positive leading edge of GBM and very few cells expressed ZDHHC23 in the CD44-positive necrotic region of GBM (Additional file 1: Figure S2a). We subsequently determined the levels of ZDHHC18 and ZDHHC23 in several validated proneural and mesenchymal GSCs and neural progenitor cell models. The expression of ZDHHC18 was markedly increased in the mesenchymal GSCs, whereas that of ZDHHC23 was associated with the proneural GSCs (Fig. 3d). To further approve that ZDHHC18 is a mesenchymal GSCs marker and ZDHHC23 is a marker for proneural GSCs, we also used SOX2 and YKL40 to identify and verify the ZDHHC18-positive and ZDHHC23-positive GBM cells, respectively. The results of WB also indicated ZDHHC18-positive GBM cells were higher expression of YKL40, and ZDHHC23-postive GBM cells highly expressed OLIG2 protein (Additional file 1: Figure S2b).

The regional variations in the transcriptional signatures correlated with the vascular and hypoxic features, and both vascular signatures and hypoxia were significantly inversely-correlated with patient survival [27, 28]. The gene expression profiling for vascular and hypoxic markers confirmed that ZDHHC18 expression was predominantly associated with hypoxia and microvascular regions in mesenchymal cells, and ZDHHC23 expression was predominantly associated with vascular regions in proneural-enriched cells (Fig. 3e). To determine the functional role for differential DHHC expression in GSCs, we investigated the viability of GSCs and neural precursors under stress, including hypoxia and nutrient restriction (Fig. 3f and Additional file 1: Figure S3a). The mesenchymal GSCs exhibited lower sensitivity to stress and preferentially survived the imposed stress, whereas almost all the neural precursors and proneural GSCs died. Consistent with these outcomes, more apoptotic cells were observed in proneural GSCs under stress conditions than in mesenchymal GSCs (Additional file 1: Figure S3 b and c). Thus, stress conditions similar to those found in the pseudopallisading necrotic regions may select for mesenchymal glioma cells with high expression of ZDHHC18. Under stress, ZDHHC23 depletion or ZDHHC18 upregulation in proneural GSCs could increase the cell survival. In contrast, targeting ZDHHC18 expression or ZDHHC23 upregulation in mesenchymal GSCs under stress reduced the cell survival, suggesting that ZDHHC18 rather than ZDHHC23 expression might be essential under stress conditions.

Because the GBM transcriptional groups are plastic, these results prompted us to speculate that differential DHHC expression might regulate the cellular transformation between proneural and mesenchymal GSCs. In proneural GSCs, ZDHHC23 depletion or ZDHHC18 upregulation could reduce the expression levels of the proneural markers and increase the levels of mesenchymal markers, to varying degrees (Fig. 3g). Notably, the gene expression profiles of ZDHHC23-depleted and ZDHHC18-overexpressing proneural GSCs under stress were observed to be similar to that of mesenchymal GSCs. These results were also obtained in mesenchymal GSCs, demonstrating that ZDHHC18 depletion and ZDHHC23 upregulation could induce the mesenchymal-to-proneural transition (Fig. 3h). Overall, we demonstrated that ZDHHC18 and ZDHHC23 target different subsets of GSCs in specific niches, with the different DHHC expression profiles potentially able to regulate the plasticity of the cellular state of a subtype.

### BMI1 constitutes an important effector in both proneural and mesenchymal GSCs

**Fig. 4:**
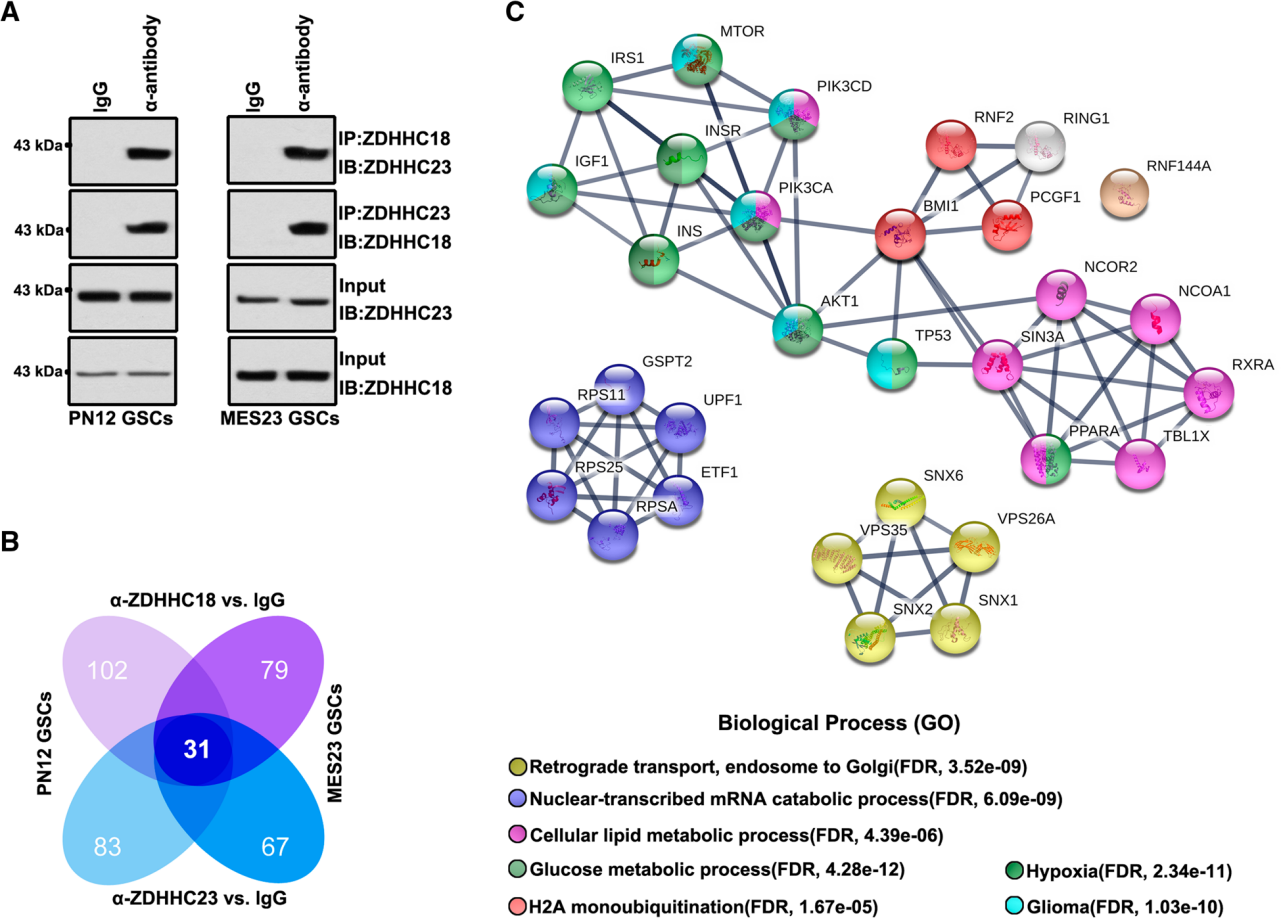
BMI1 constitutes a potential interaction partner in the ZDHHC18/ZDHHC23 protein interaction network in proneural and mesenchymal glioma stem cells (GSCs). **a, b** Lysates from proneural GSCs (PN12) and mesenchymal GSCs (MES23) were subjected to immunoprecipitation using anti-ZDHHC18 and anti-ZDHHC23 antibodies, respectively, and then (**a**) immunoblotted with anti-ZDHHC18 and anti-ZDHHC23 antibodies, or (**b**) the IP-protein complex was subjected to LC/MS-MS analysis. Venn diagrams showing the overlaps between specific peaks of the various IP-protein complexes. **c** Protein–protein interaction networks indicated by STRING software showing the overlapping peaks in both the proneural and mesenchymal GSCs after immunoprecipitation with anti-ZDHHC18 and anti-ZDHHC23 antibodies, respectively. The shared regions mostly converged on 31 protein targets, generally associated with retrograde transport, nuclear-transcribed mRNA catabolic process, cellular lipid, and glucose metabolic processes, H2A monoubiquitination, hypoxia microenvironment, and glioma development

Although ZDHHC18 and ZDHHC23 expression patterns differed between the proneural and mesenchymal GSCs, these two proteins were partly co-localized in both cell types, indicating their likely association. Accordingly, the interaction between ZDHHC18 and ZDHHC23 was confirmed by immunoprecipitation analysis of proneural and mesenchymal GSCs (Fig. 4a). Next, we interrogated the complexes of ZDHHC18 and ZDHHC23 with other proteins in the proneural and mesenchymal GSCs using anti-ZDHHC18 and ZDHHC23 antibodies, respectively, and the components of the complexes were identified using liquid chromatography/tandem mass spectrometry (LC/MS-MS). We analyzed the overlapping peaks in both the proneural and mesenchymal GSCs and identified those that were unique to a particular subtype of GSCs or shared between the proneural and mesenchymal GSCs (Fig. 4b). The protein complexes of ZDHHC18 and ZDHHC23 in the same subtype of GSCs revealed over 60% subtype-specific protein targets, indicating distinct DHHC expression profiles or functions in GSCs residing in different regions. However, the shared regions mostly converged on 31 target proteins that are generally associated with retrograde transport, nuclear-transcribed mRNA catabolic process, cellular lipid and glucose metabolic processes, H2A monoubiquitination, hypoxia microenvironment, and glioma development (Fig. 4c).

**Fig. 5:**
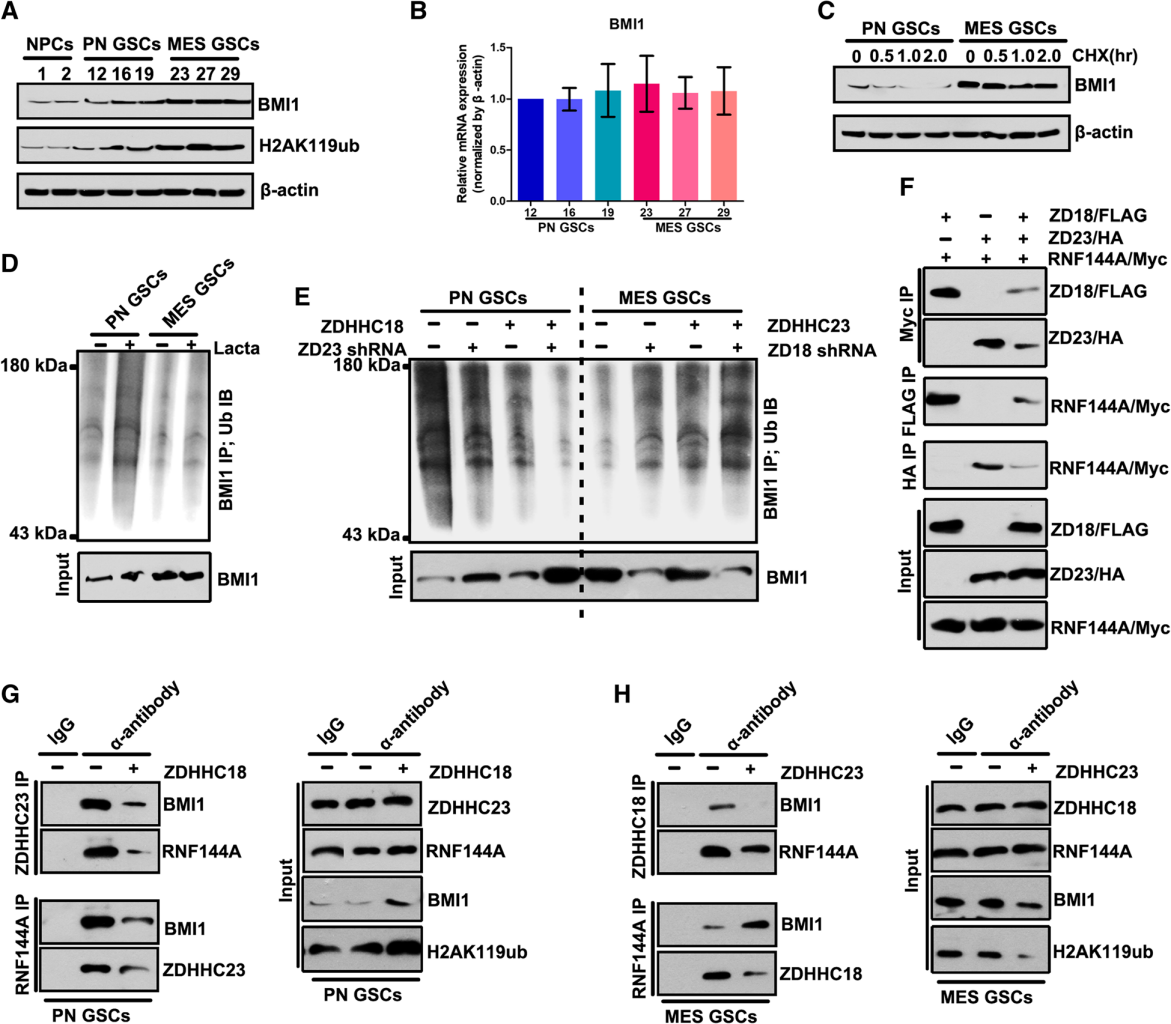
ZDHHC18 and ZDHHC23 regulate BMI1 polyubiquitination. **a** Expression levels of BMI1 and H2AK119Ub detected by western blot analysis in neural progenitor cells (NPC1 and NPC2), proneural glioma stem cells (GSCs) (PN12, PN16, and PN19), and mesenchymal GSCs (MES23, MES27, and MES29). β-actin was used as a loading control. **b** mRNA levels of BMI1 were detected by RT-PCR analysis in the proneural GSCs (PN12, PN16, and PN19) and mesenchymal GSCs (MES23, MES27, and MES29). β-actin was used as a loading control. Data are presented as means ± SEM. **c** BMI1 protein expression in PN12 and MES23 GSCs after cycloheximide treatment (CHX, 50 μM) for the indicated times. **d, e** Immunoprecipitation followed by immunoblotting was performed for determining BMI1 polyubiquitination in PN12 and MES23 GCSs in the presence and absence of lactacystin treatment for 5 h (Lacta, 10 μM) (**d**) or transfected with the indicated plasmids in the presence of lactacystin treatment for 5 h (Lacta, 10 μM) (**e**). **f** Lysates from 293 T cells expressing the FLAG-ZDHHC18, HA-ZDHHC23, and Myc-RNF144A were subjected to immunoprecipitation, followed by immunoblotting with anti-FLAG, anti-HA, and anti-Myc antibodies. **g, h** Lysates from PN12 (**g**) or MES23 (**h**) GSCs transfected with the indicated plasmids were subjected to immunoprecipitation with anti-ZDHHC23 (**g**), anti-ZDHHC18 (**h**), or anti-RNF144A antibody (**g** and **h**), followed by immunoblotting with anti-BMI1, anti-RNF144A, anti-ZDHHC18, and anti-ZDHHC23 antibodies

Notably, the BMI1 protein complex was located in a key position for the ZDHHC18- ZDHHC23 protein network. The BMI1 protein complex plays an important role in cellular lipid and glucose metabolic processes and under hypoxic microenvironment [29, 30]. Thus, we surmised that ZDHHC18 and ZDHHC23 might regulate cellular survival in areas of low oxygen and nutrient availability, through the BMI1 complex. We first investigated BMI1 in the validated proneural and mesenchymal GSCs and in neural progenitor cell models. The expression of BMI1 and its target, H2AK119Ub, was markedly increased in mesenchymal GSCs (Fig. 5a), similar to the expression pattern of ZDHHC18 but unlike that of ZDHHC23 (Fig. 3d). However, there was no difference in the mRNA level of BMI1 between the proneural and mesenchymal GSCs (Fig. 5b), suggesting that BMI1 might be regulated post-transcriptionally in these GSC subtypes. The inhibition of translation with cycloheximide showed that the levels of BMI1 were unchanged in the mesenchymal GSCs but markedly reduced in the proneural GSCs (Fig. 5c). Conversely, the level of polyubiquitinated BMI1 was strongly increased in the proneural GSCs after treatment with lactacystin, a proteasome inhibitor, but accumulated to low levels in the mesenchymal GSCs under the same conditions (Fig. 5d), suggesting that ubiquitin-mediated proteolysis maintains the stability of BMI1 in the different GSC subtypes. Depletion of ZDHHC23 or over-expression of ZDHHC18 in the proneural GSCs reduced the content of polyubiquitinated BMI1 after inhibition of proteolysis and concordantly increased the expression of BMI1 protein (Fig. 5e and Additional file 1: Figure S2). Moreover, the depletion of ZDHHC18 or up-regulation of ZDHHC23 increased the level of polyubiquitinated BMI1 and reduced the expression of BMI1.

The BMI1 E3 ligase RNF144A constitutes one of the components of the ZDHHC18- ZDHHC23 protein network. Immunoprecipitation analysis of 293 T cells transfected with Flag-tagged ZDHHC18, HA-tagged ZDHHC23, and Myc-tagged RNF144A confirmed that ZDHHC18 and ZDHHC23 competitively interacted with RNF144A (Fig. 5f). In proneural GSCs, the interaction between ZDHHC23 and RNF144A was also identified whereas ZDHHC18 over-expression was found to reduce this interaction but increased the level of BMI1 protein (Fig. 5g). ZDHHC18 could also interact with RNF144A in the mesenchymal GSCs, with ZDHHC23 over-expression reducing this interaction and also down-regulating the level of BMI1 protein (Fig. 5h). Thus, these results suggested that ZDHHC23 could recruit RNF144A and BMI1 to increase the level of polyubiquitinated BMI1 in the proneural GSCs. ZDHHC18 could also interact with RNF144A, whereas ZDHHC18 hindered the interaction of RNF144A and BMI1 to reduce the level of polyubiquitinated BMI1 in mesenchymal GSCs.

### BMI1 inhibitor is effective against the mesenchymal subgroup of GBM

**Fig. 6:**
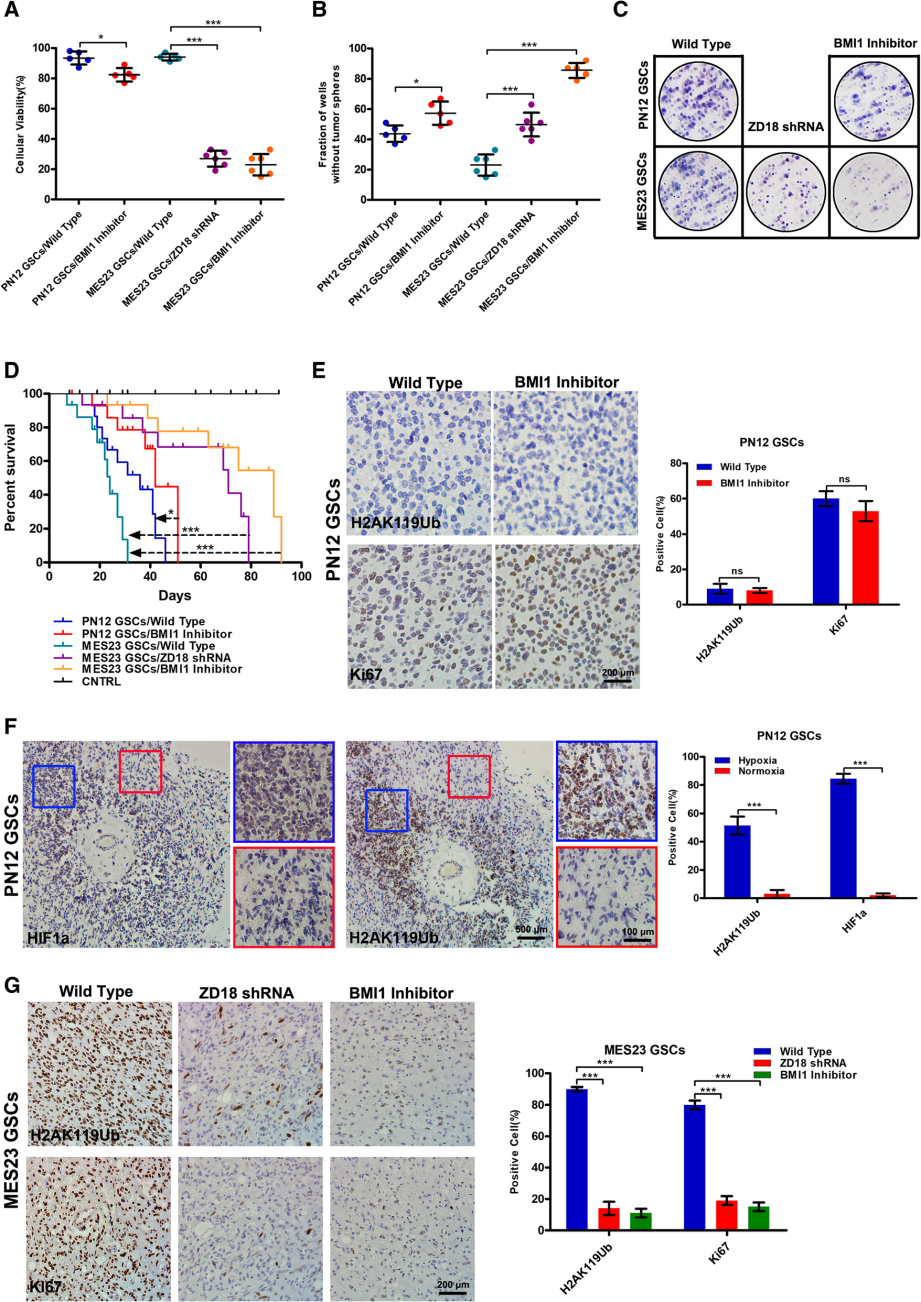
BMI1 inhibitor (PTC596) is effective against the mesenchymal subgroup of glioblastoma (GBM). **a-c** Cell viability (**a**), tumorsphere formation (**b**), and foci formation (**c**) was determined for proneural (PN12) and mesenchymal (MES23) glioma stem cells (GSCs) transfected with the indicated plasmids in the presence or absence of PTC596 (10 μM) treatment for 24 h (**a**), 72 h (**b**), or 2 weeks (**c**) . Data are presented as means ± SEM (*, *p* < 0.05; ***, *p* < 0.001). **d** Kaplan–Meier survival curve showing the survival of NOD SCID gamma mice injected with 500 PN12 or 500 MES23 (or shRNA-ZDHHC18 MES23) GSCs with or without PTC596 treatment (*n* = 15; *, *p* < 0.05; ***, *p* < 0.001). **e** Immunohistochemistry analysis for H2AK119Ub and Ki-67 in sections from the xenografts of intracranially implanted PN12 GSCs. Quantification of H2AK119Ub and Ki67 positive cells in three fields is presented. Error bars represent the SEM (*ns*, not significant). Scale bar, 200 μm. **f** Immunohistochemistry analysis for HIF1α and H2AK119Ub in sections from the xenografts of intracranially implanted PN12 GSCs. Blue box, hypoxic region; red box, normoxic region. Scale bar: (left image) 500 μm; (right image, inset box) 100 μm (white). Quantification of HIF1α and H2AK119Ub positive cells in three fields is presented. Error bars represent the SEM (***, *p* < 0.001). **g** Immunohistochemistry analysis for H2AK119Ub and Ki-67 in sections from the xenografts of intracranially implanted MES23 GSCs transfected with the indicated plasmids with or without PTC596 treatment. Quantification of H2AK119Ub and Ki67 positive cells in three fields is presented. Error bars represent the SEM (***, *p* < 0.001). Scale bar, 200 μm

The presence of mesenchymal GSCs is closely associated with the recurrence of glioma after radiation or chemotherapy [31, 32]. To leverage our findings for clinical application, we examined the sensitivity of GSC subtypes to PTC596, a BMI1 inhibitor. The mesenchymal GSCs displayed preferential sensitivity to PTC596 treatment, similar to the effect of ZDHHC18 depletion (Fig. 6a). Moreover, the inhibitory effect of PTC596 was higher in the mesenchymal GSCs than in the proneural GSCs. The self-renewal and foci formation of GSCs revealed the sensitivity of mesenchymal GSCs to PTC596 (Fig. 6b and c).

Next, we examined the therapeutic effects of PTC596 on mice bearing intracranial tumors derived from proneural or mesenchymal GSCs. The inhibition of BMI1 activity was not able to more efficiently extend the survival rate of animals implanted with proneural GSCs compared to that in animals implanted with mesenchymal GSCs (Fig. 6d). In contrast, knockdown of ZDHHC18 or PTC596 injection obviously resulted in the inhibition of tumor growth and extended the survival rate of all animals that were implanted with mesenchymal GSCs (Fig. 6d). The results of immunohistochemical analysis of intracranial tumors derived from proneural GSCs further confirmed that in general, no differences were observed in the incidence of H2AK119Ub and Ki67-positive cells between the PTC596-treated and non-treated groups (Fig. 6e). Notably, the plasticity of cell state transitions toward the proneural-to-mesenchymal subgroup was also confirmed, as indicated by the observation that the expression level of H2AK119Ub was somewhat higher in the partial hypoxic region than that in the normoxic region (Fig. 6f). Moreover, the knockdown of ZDHHC18 or PTC596 injection obviously reduced the prevalence of H2AK119Ub and Ki67-positive cells in tumors implanted with mesenchymal GSCs, compared to that in the non-treated group (Fig. 6g). These in vivo results indicated that the depletion of ZDHHC18 or inhibition of BMI1 might serve as an effective treatment strategy for the mesenchymal subtype GBM-associated stress microenvironment.

## Discussion

**Fig. 7:**
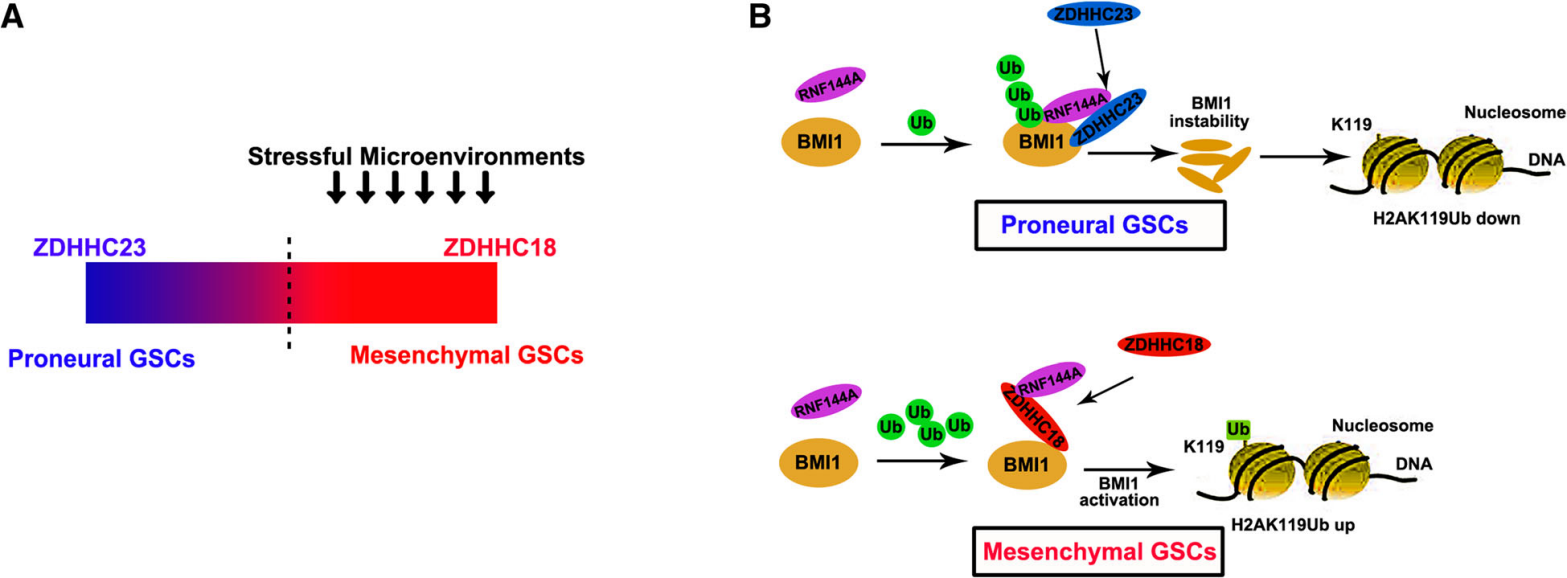
Schematic diagram. **a** Schematic diagram showing induction of the mesenchymal-to-proneural transition by the depletion of ZDHHC18 and the upregulation of ZDHHC23. Heterogeneous distribution of glioma stem cells (GSCs) occurs in different types of niches and the transcriptional groups of glioblastoma are plastic. The response to stress might be chosen based on the dependence on DHHC proteins, in which cellular survival is promoted by ZDHHC18 under nutrient scarcity and low oxygen stress. **b** Schematic diagram showing that the ubiquitin-mediated proteolysis maintains BMI1 protein stability in different GSC subtypes. In the proneural GSCs, ZDHHC23 recruits RNF144A for polyubiquitination of BMI1. However, ZDHHC18 hinders the association of RNF144A and BMI1 to reduce the level of polyubiquitinated BMI1 in the mesenchymal GSCs

An increasing number of studies have shown that dysfunction or dysregulation of the proteins of the DHHC family plays a pivotal role in tumorigenesis, particularly in the progression and malignant development of gliomas [22, 33–35]. Herein, we found that the response to stress is dependent on the DHHC proteins; in particular, cellular survival is promoted by ZDHHC18 under nutrient scarcity and low oxygen conditions (Fig. 7a). To enhance the level of polyubiquitinated BMI1 in proneural GSCs, BMI1 and RNF144A can be recruited by ZDHHC23. However, ZDHHC18 affects the association of BMI1 and RNF144A and competitively interacts with RNF144A to decrease the level of polyubiquitinated BMI1 in mesenchymal GSCs (Fig. 7b). These findings support the model wherein the survival of mesenchymal GSCs is promoted by ZDHHC18 under stressful microenvironment through an increase in the stability of BMI1.

Cancer and DHHC enzymes are closely associated; for example, copy number variation of a chromosome region including the gene of ZDHHC11 is observed in lung and bladder cancers [36], and decreased expression of ZDHHC2 has been found in colorectal cancers and gastric adenocarcinoma [37]. In addition, upregulation of ZDHHC9 has been found in colorectal tumors [38]. Moreover, a number of DHHC enzymes appear to function as key players underlying tumorigenesis in gliomas. The up-regulation of ZDHHC5, as an oncogenic factor, has been reported in p53-mutant gliomas [22]. Moreover, the gene encoding ZDHHC17 is located in the chromosomal region containing a potential oncogene for glioma and ZDHHC17 protein can interact with MAP2K4 to regulate the development and progression of malignant glioma and stimulate JNK/p38 (our unpublished data).

Although human gliomas can be characterized at the molecular level, the importance of changes in the expression of given gene cannot directly be ascribed to any underlying biological function [39, 40]. Herein, we studied the function of DHHC family proteins in gliomas and found that ZDHHC18 and ZDHHC23 are preferentially expressed in GBMs in comparison to LGGs. The high expression level of *ZDHHC18* relates to the mesenchymal molecular phenotype and that of ZDHHC23 is related to the proneural phenotype. In addition, poor prognosis in patients with glioma associates with high co-expression levels of ZDHHC18 and ZDHHC23. In contrast, low levels of *ZDHHC18* and *ZDHHC23* mRNA are associated with other positive prognostic markers including the loss of *TERT,* mutations of *ATRX* and *IDH1*, 1p/19q codeletion, and methylation of *MGMT*. Notably, the stem cell state of GBM might be reflected by the expression levels of ZDHHC23 and ZDHHC18, as these DHHC proteins can be expressed in regular neural stem cells. Moreover, whereas the roles of ZDHHC18 and ZDHHC23 are not biochemically interchangeable, they might permit plasticity of these cells under different microenvironmental conditions. The upregulation of ZDHHC18 or the depletion of ZDHHC23 to different degrees in the proneural GSCs could promote the expression of mesenchymal markers and decrease the expression of proneural markers. Furthermore, the expression profile of ZDHHC18-overexpressing and ZDHHC23-depleted proneural GSCs under stress was similar to that of mesenchymal GSCs. It was also observed that in the mesenchymal GSCs, the mesenchymal-to-proneural transition could be turned on by the upregulation of ZDHHC23 and the depletion of ZDHHC18. These results support a model wherein GSCs can be found in niches with different usages of ZDHHC18 and ZDHHC23, and cellular fate transformation between mesenchymal and proneural GSCs might be regulated by different expression profiles of DHHC proteins.

Proteins of the Polycomb (PcG) family are important for cell cycle regulation and maintenance of cellular identity [41, 42], with functions underlining their involvement in carcinogenesis in humans. In the subsets of gliomas, the *BMI1* gene is aberrant at the chromosomal level, positively contributing to the pathogenesis of brain tumors [18, 43]. In particular, BMI1 might be required for the maintenance and renewal of cancer initiating stem cells and might be involved in the growth of GBMs [18, 44]. The locus of *INK4A/ARF*, which encodes the two tumor suppressor proteins, p14ARF and p16INK4A, constitutes a major target of stem cell activities and BMI1 oncogenic proliferation [20, 45]. It has been reported that the activation signature of BMI1 negatively correlates with a proneural microenvironment signature and positively correlates with classical or mesenchymal microenvironment signatures [21, 46]. However, the activity of BMI1 does not correlate with the levels of *BMI1* mRNA and the levels of BMI1 protein are higher in mesenchymal than in proneural tumors.

RNF144A, an E3 ligase involved in post-translational regulation of BMI1, provides a novel mechanism for BMI1 regulation in GBM. Indeed, ubiquitin-mediated proteolysis maintains the stability of BMI1 protein in different GSC subtypes. We observed that the level of nonubiquitinated BMI1 protein was increased upon inhibition of proteolysis, and that of polyubiquitinated BMI1 was decreased by over-expression of ZDHHC18 or depletion of ZDHHC23 in proneural GSCs. In addition, the upregulation of ZDHHC23 or depletion of ZDHHC18 could decrease the level of nonubiquitinated BMI1 protein and increase the level of polyubiquitinated BMI1. The results of immunoprecipitation assays using 293 T cells transfected with Myc-tagged RNF144A, HA-tagged ZDHHC23, and Flag-tagged ZDHHC18 confirmed that ZDHHC23 and ZDHHC18 competitively interact with RNF144A, the BMI1 E3 ligase, which should, therefore, constitute a component of the protein networks of ZDHHC18 and ZDHHC23. As revealed by an inspection of the cBioPortal database, many types of cancers, such as lung, prostate, breast, and uterine cancer, have amplifications or mutations in RNF144A [47, 48]. In addition, the interaction between RNF144A and ZDHHC23 in the proneural GSCs could be identified and the over-expression of ZDHHC18 could enhance the level of BMI1 yet decrease the interaction. Moreover, the over-expression of ZDHHC23 decreased the interaction whereas ZDHHC18 interacted with RNF144A in the mesenchymal GSCs, accompanying down-regulation of BMI1 levels. The interaction between ZDHHC23 and RNF144A was also identified in the proneural GSCs, with the overexpression of ZDHHC18 reducing this interaction but increasing the level of BMI1. ZDHHC18 itself could also interact with RNF144A, although this hindered the association between BMI1 and RNF144A to decrease the level of polyubiquitinated BMI1 in the mesenchymal GSCs.

The mesenchymal-to-proneural transition could be induced by the depletion of ZDHHC18 and the upregulation of ZDHHC23. Notably, the plasticity of cell state transitions toward the proneural to-mesenchymal subgroup could be confirmed; for example, the expression level H2AK119Ub was lower in the normoxic region than that in the hypoxic region. Considering that the presence of mesenchymal GSCs is closely related to the recurrence of glioma after chemo- and radiotherapy, the results of the present study suggest that the inhibition of BMI1 or the depletion of ZDHHC18 might serve as an effective strategy for treatment of the stress microenvironment related to the mesenchymal subtype of GBM.

## Conclusion

In summary, increasing evidence has shown that heterogeneous distribution of GSCs occurs in different niches and the transcriptional groups of GBM are plastic. It could be demonstrated that the importance of the expression pattern of BMI1 is facilitated by the family of DHHC proteins with regard to plasticity of the cell state transition for GSCs of GBM. Therefore, the DHHC family proteins may represent prime molecular targets in the therapy of patients with glioma as these proteins can orchestrate essential aspects of cancer stem cell biology.

## Additional file

Additional file 1: **Table S1.** Correlation of ZDHHC18 or ZDHHC23 expression in human glioma patients with different clinicopathological features. **Figure S1.** The subtype-characterized GBMc cells (PN12, PN16, PN19, MES23, MES27, and MES29) were isolated from surgical specimens and were functionally validated. **Figure S2.** Association of expression levels ofZDHHC18 (or ZDHHC23) and stem cell marker. **Figure S3.** ZDHHC18 and 23 target different subsets of GSCs in specific niches. **Figure S4.** Expression levels of ZDHHC18 and ZDHHC23 detected by western blot analysis in proneural (PN12) and mesenchymal (MES23) GSCs transfected with indicated plasmids. (PDF 1080 kb)

## Abbreviations

ARTX: X-linked α-thalassemia retardation protein
DHHC: aspartate-histidine-histidine-cysteine
GBM: Glioblastmas
IDH: Isocitrate dehydrogenase
MGMT: O^6^-methylguanine-DNA methyltransferase
qRT-PCR: Quantitative reverse transcription polymerase chain reaction
RNF144A: Ring Finger protein 144A
TERT: Telomerase reverse transcriptase
