# Supplementary material for: DHHC protein family targets different subsets of glioma stem cells in specific niches
Source: J Exp Clin Cancer Res. 2019 Jan 18;38:25. doi: 10.1186/s13046-019-1033-2 (PMC6339410; doi:10.1186/s13046-019-1033-2)
Supplement: Supplementary file 1 — Table S1. Correlation of ZDHHC18 or ZDHHC23 expression in human glioma patients with different clinicopathological features. Figure S1. The subtype-characterized GBMc cells (PN12, PN16, PN19, MES23, MES27, and MES29) were isolated from surgical specimens and were functionally validated. Figure S2. Association of expression levels ofZDHHC18 (or ZDHHC23) and stem cell marker. Figure S3. ZDHHC18 and 23 target different subsets of GSCs in specific niches. Figure S4. Expression levels of ZDHHC18 and ZDHHC23 detected by western blot analysis in proneural (PN12) and mesenchymal (MES23) GSCs transfected with indicated plasmids. (PDF 1080 kb) [file 13046_2019_1033_MOESM1_ESM.pdf]

Table S1. Correlation of ZDHHC18 or ZDHHC23 expression in human glioma patients with different clinicopathological features.

Variable		ZDHHC18		*p* Value	ZDHHC23		*p* Value
		High	Low		High	Low	
Age	≥45	27	21	>0.05	31	17	>0.05
	<45	18	24		20	22	
Gender	Male	32	22	>0.05	21	32	>0.05
	Female	27	19		20	26	
KPS	≥80	32	31	<0.05	27	42	>0.05
	<80	21	6		13	14	
Grade	Ⅰ/Ⅱ	8	16	<0.01	4	32	<0.01
	Ⅲ	23	7		12	18	
	Ⅳ	31	5		22	2	
Subtype	Neural	7	12	<0.01	2	17	<0.01
	Proneural	6	8		18	3	
	Classic	12	13		9	9	
	Mesenchymal	29	3		4	28	
IDH1	Wild-Type	42	11	<0.01	32	11	>0.05
	Mutant	13	24		10	27	
MGMT	Methylated	16	36	>0.05	26	26	>0.05
	Unmethylated	13	25		29	19	
1p/19q	Codeletion	8	32	<0.01	12	28	<0.01
	Non-codeletion	33	17		37	23	
TERT	Wild-Type	19	7	<0.01	23	3	<0.05
	Mutant	23	41		43	21	
ATRX	Wild-Type	43	11	<0.01	32	22	<0.01
	Mutant	19	27		11	35	

p values were determined by the Chi-square and Fisher's exact tests.

Figure S1

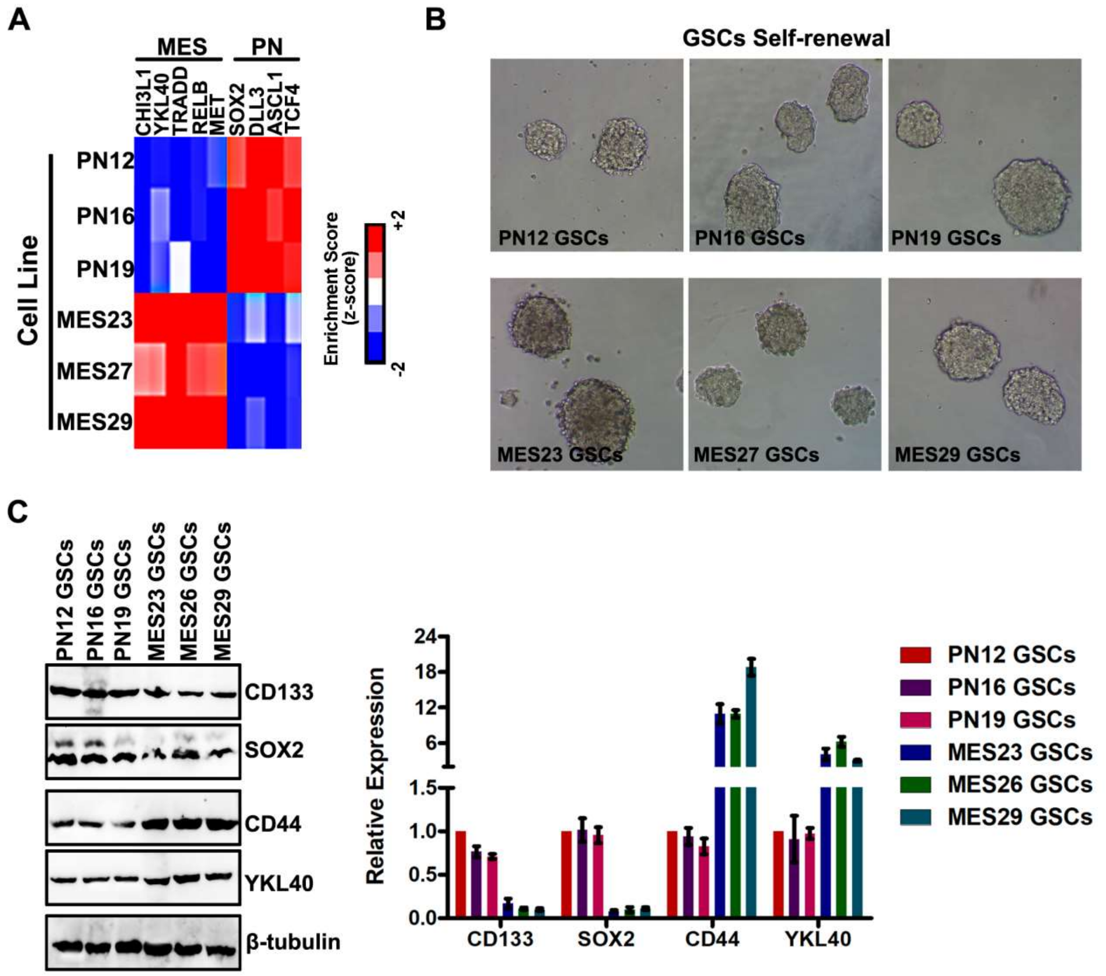

Figure S1. The subtype-characterized GBMc cells (PN12, PN16, PN19, MES23, MES27, and MES29) were isolated from surgical specimens and were functionally validated. A, Heat map showing the molecular subtype marker expression in proneural GBM cells (PN12, PN16 and PN19), and mesenchymal GBM cells (MES23, MES27 and MES29). Proneural marker: SOX2, DLL3, ASCL1 and TCF4; mesenchymal marker: CHI3L1, YKL40, TRADD, RELB and MET. Z-scores were calculated from the ΔCt values obtained in the qPCR analysis. B, GSCs self-renewal of proneural GBM cells (PN12, PN16 and PN19), and mesenchymal GBM cells (MES23, MES27 and MES29). C, GSC marker (CD133, SOX2, CD44 and YKL40) in proneural GSCs (PN12, PN16 and PN19), and mesenchymal GSCs (MES23, MES27 and MES29). β-tubulin was used as the loading control. Data represent the mean of at least three independent experiments ± SD.

Figure S2

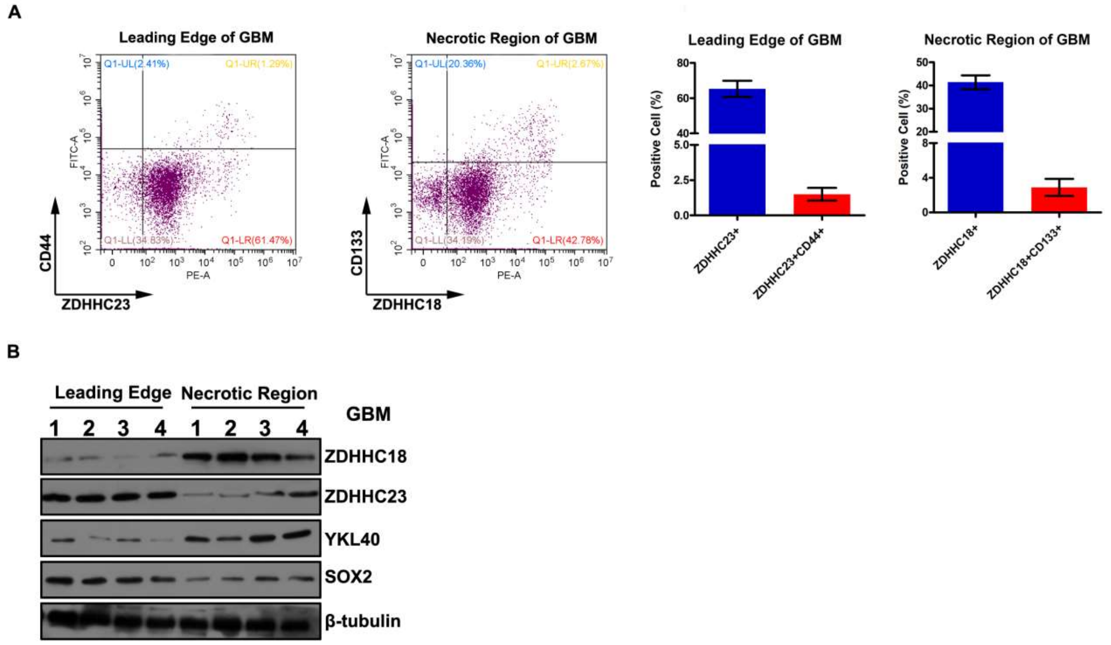

Figure S2. Association of expression levels ofZDHHC18 (or ZDHHC23) and stem cell marker. A, Results of flow cytometry analysis showing the relationship between the expression of ZDHHC18 (or ZDHHC23) and stem cell marker CD133 (or CD44) in the leading edge and neurotic regions of the GBM samples. Quantification of ZDHHC23, ZDHHC23/CD44, ZDHHC18 and ZDHHC18/CD133 positive cells in three fields is presented. Error bars represents the SEM. B, Expression levels of ZDHHC18 (or ZDHHC23) and stem cell marker YKL40 (or SOX2) detected by western blot analysis in the leading edge and necrotic region of GBM. β-tubulin was used as a loading control for the western blot analysis.

Figure S3

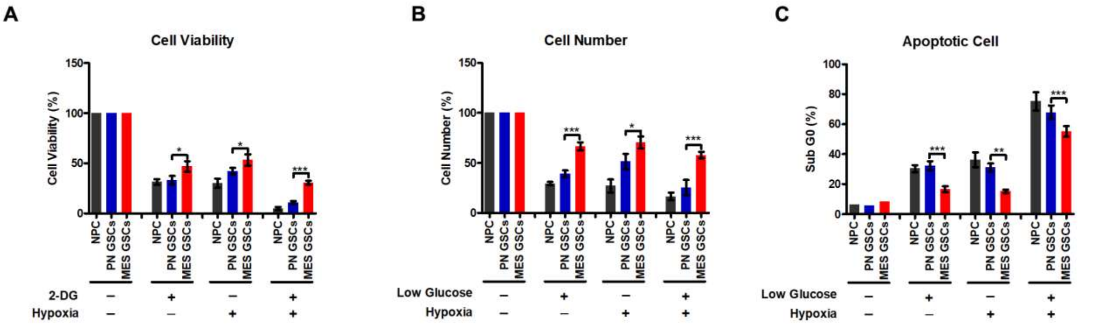

Figure S3. ZDHHC18 and 23 target different subsets of GSCs in specific niches. A, Cell viability of proneural GSCs (PN12, PN16 and PN19), and mesenchymal GSCs (MES23, MES27 and MES29) under baseline, 2-DG, hypoxia, or conditions with a combination of these stresses. B and C, Numbers of survival cell and apoptotic cell in proneural GSCs (PN12, PN16 and PN19), and mesenchymal GSCs (MES23, MES27 and MES29) under baseline, low-glucose, hypoxia, or conditions with a combination of these stresses. Data are presented as means ± SEM (*, $p < 0.05$; **, $p < 0.01$; ***, $p < 0.001$).

Figure S4

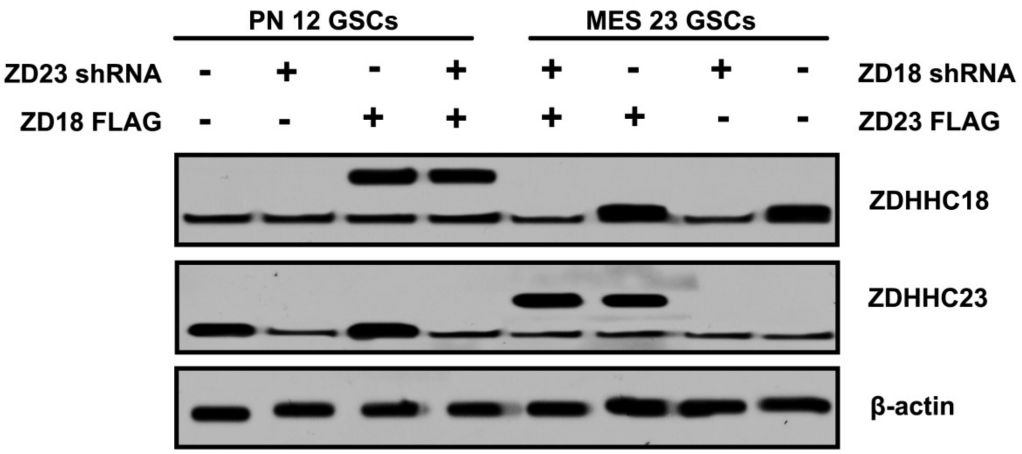

Figure S4. Expression levels of ZDHHC18 and ZDHHC23 detected by western blot analysis in proneural (PN12) and mesenchymal (MES23) GSCs transfected with indicated plasmids. β-actin was used as a loading control for the western blot analysis.
